# Development of a new oral poliovirus vaccine for the eradication end game using codon deoptimization

**DOI:** 10.1038/s41541-020-0176-7

**Published:** 2020-03-20

**Authors:** Jennifer L. Konopka-Anstadt, Ray Campagnoli, Annelet Vincent, Jing Shaw, Ling Wei, Nhien T. Wynn, Shane E. Smithee, Erika Bujaki, Ming Te Yeh, Majid Laassri, Tatiana Zagorodnyaya, Amy J. Weiner, Konstantin Chumakov, Raul Andino, Andrew Macadam, Olen Kew, Cara C. Burns

**Affiliations:** 1 Division of Viral Diseases, Centers for Disease Control and Prevention, Atlanta, GA USA; 2 IHRC, Inc., contracting agency to the Division of Viral Diseases, Centers for Disease Control and Prevention, Atlanta, GA USA; 3 Cherokee Nation Assurance, contracting agency to the Division of Viral Diseases, Centers for Disease Control and Prevention, Atlanta, GA USA; 4 National Institute for Biological Standards and Control, Hertfordshire, UK; 5 Department of Microbiology and Immunology, University of California, San Francisco, CA USA; 6 Center for Biologics Evaluation and Research, US Food and Drug Administration, Silver Spring, MD USA; 7 Bill and Melinda Gates Foundation, Seattle, WA USA; 8 Global Virus Network Center of Excellence, Baltimore MD, USA

**Keywords:** Policy and public health in microbiology, Vaccines, Live attenuated vaccines, Virology

## Abstract

Enormous progress has been made in global efforts to eradicate poliovirus, using live-attenuated Sabin oral poliovirus vaccine (OPV). However, as the incidence of disease due to wild poliovirus has declined, vaccine-derived poliovirus (VDPV) has emerged in areas of low-vaccine coverage. Coordinated global cessation of routine, type 2 Sabin OPV (OPV2) use has not resulted in fewer VDPV outbreaks, and continued OPV use in outbreak-response campaigns has seeded new emergences in low-coverage areas. The limitations of existing vaccines and current eradication challenges warranted development of more genetically stable OPV strains, most urgently for OPV2. Here, we report using codon deoptimization to further attenuate Sabin OPV2 by changing preferred codons across the capsid to non-preferred, synonymous codons. Additional modifications to the 5′ untranslated region stabilized known virulence determinants. Testing of this codon-deoptimized new OPV2 candidate (nOPV2-CD) in cell and animal models demonstrated that nOPV2-CD is highly attenuated, grows sufficiently for vaccine manufacture, is antigenically indistinguishable from Sabin OPV2, induces neutralizing antibodies as effectively as Sabin OPV2, and unlike Sabin OPV2 is genetically stable and maintains an attenuation phenotype. In-human clinical trials of nOPV2-CD are ongoing, with potential for nOPV strains to serve as critical vaccine tools for achieving and maintaining polio eradication.

## Introduction

Once a disease feared worldwide, polio is nearing eradication with only three countries remaining that have never interrupted wild poliovirus (WPV) transmission^1,2^. Sabin oral poliovirus vaccine (OPV) and Salk inactivated poliovirus vaccine (IPV) have been crucial to the eradication effort. While vaccination has played a central role in the dramatic decline of poliomyelitis cases, unexpected challenges have become apparent, particularly associated with use of Sabin OPV^3^. Sabin OPV is a live vaccine containing attenuated strains of all three poliovirus serotypes. Unlike IPV, OPV strains replicate in the gut and induce the mucosal immunity necessary not only to limit poliovirus infection and disease but also to interrupt person-to-person transmission. OPV is inexpensive, easy to administer, and has been the centerpiece in the global eradication program.

However, it has become evident that Sabin OPV strains are genetically unstable. During replication in the human gut, the attenuated strains can evolve and regain virulence through reversion of key attenuating mutations^4^. Particularly in areas of low-vaccine coverage, where OPV strains have the opportunity to replicate unchecked and transmit for longer durations, this genetic instability can lead to circulating vaccine-derived polioviruses (cVDPVs)^5–7^, with type 2 (cVDPV2) being more prone to reversion^8–10^. Accordingly, a global coordinated withdrawal of trivalent Sabin OPV and switch to bivalent OPV (containing only type 1 and 3 Sabin strains) occurred in 2016^11^. Despite the withdrawal of Sabin OPV2, cVDPV outbreaks have continued to emerge in high-risk areas^12–15^.

Polio will be fully eradicated only when all sources of the virus, including both WPV and VDPV, are eliminated. Despite the undeniable importance of routine and mass administration of Sabin OPV in preventing millions of cases of paralytic poliomyelitis, achieving complete eradication necessitates the development of new OPV strains with less inherent risk. While replacement of Sabin OPV with IPV or other non-infectious vaccine alternatives would confer individual protection from paralytic disease, the lack of live replicating virus has limited capacity to induce the gut immunity required to impede poliovirus transmission in developing country settings. Much is now understood regarding the biology underlying attenuation mechanisms of the Sabin OPV strains, as well as their instability^16–19^. In 2011, the New OPV Consortium (nOPV Consortium) was established and tasked with the development of improved OPV strains based on innovative strategies.

We used codon deoptimization of the capsid region, combined with stabilization of known attenuation determinants in the Sabin 5′ untranslated region (UTR), to engineer a nOPV2 strain that is genetically stable. Our previous work demonstrated that synonymous codon substitutions increasing CpG and UpA dinucleotide pairs in the capsid region reduce replicative fitness of both WPV and Sabin OPV strains^20,21^. This strategy is based on CpG and UpA dinucleotide desuppression and the biology of codon usage bias, which is observed in all biological systems, including viruses^22–27^. Naturally preferred codons can be replaced with “unpreferred” synonymous codons to modulate viral replicative fitness without introducing changes at the protein-coding level^28–31^.

Codon-deoptimized (CD) polioviruses have reduced replicative fitness proportional to the number of codon replacements made, with the key contribution to this phenotype being the increase in the number of dinucleotides (e.g., CpG and UpA) added^20,21^. The level of viral fitness can thereby be modulated by varying the number of engineered synonymous changes. The capsid was targeted for modification because poliovirus is known to recombine with other human enteroviruses, and often only the capsid is retained^6,32^. Securing the modifications within the capsid region safeguards the attenuated phenotype even in recombinants, preventing further spread of Sabin-derived capsid vulnerable to reversion in the field. To further improve genetic stability, our codon-deoptimized nOPV2 strain (nOPV2-CD) also features a modified 5′ UTR. This modification stabilizes a known attenuation determinant in Sabin OPV2, preventing reversion at the site which is associated with many cVDPV2^4,17,33–38^.

Here we report the preclinical evaluation of nOPV2-CD in cell and animal models, demonstrating that nOPV2-CD maintains attenuation and immunogenicity yet offers the advantage of increased genetic stability. The results of these preclinical studies have served as groundwork supporting the decision to move nOPV2-CD into human clinical trials, which are ongoing^39^. Contingent on a successful clinical trial outcome, nOPV2 could replace Sabin mOPV2 in the global stockpile, particularly as a tool to mitigate outbreaks in settings most prone to low immunization coverage and VDPV emergence, situations that likely pose a threat even post-eradication.

## Results

### Codon-deoptimized nOPV2 exhibits reduced replicative fitness

**Fig. 1:**
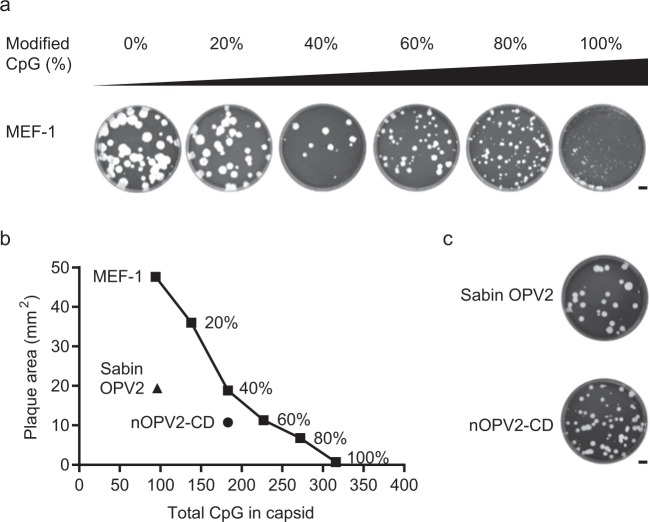
Codon-deoptimized wild- and Sabin-derived strains of type 2 poliovirus exhibit reduced replicative fitness. Type 2 WPV strain MEF-1 was codon deoptimized by modifying synonymous codons at 20 to 100% of possible CpG_2-3_ sites within the capsid region. **a** Plaque morphology of codon-deoptimized MEF-1 on HeLa cell monolayers after 65 h at 37 °C. **b** Mean plaque area of codon-deoptimized MEF-1 viruses as compared to Sabin OPV2 and nOPV2-CD on HeLa cell monolayers after 65 h at 37 °C. **c** Plaque morphology of Sabin OPV2 and nOPV2-CD on HeLa cell monolayers after 65 h at 37 °C. Representative images and measurements of *n* = 2 biologically independent replicates showing similar results shown. Scale bar = 1 cm.

Our previous work has demonstrated that synonymous codon substitutions increasing CpG_2-3_ dinucleotide pairs in the capsid region of both WPV and Sabin OPV strains reduce replicative fitness. This effect is dose-responsive, allowing for the level of replicative attenuation to be finely modulated by the extent of synonymous codon substitutions and the resulting CpG and UpA content (Fig. 1a, b). Comparing the relative fitness of Sabin OPV2 to that of type 2 wild poliovirus strain MEF-1 containing a range of codon deoptimization within the capsid region (at 20 to 100% of all possible CpG_2-3_ sites) served as a guide for the level of modification to target for nOPV2. These foundational results guided us to modify 40% of all possible CpG_2-3_ sites in the capsid region for nOPV2. The resulting plaque morphology of nOPV2-CD was similar to that of Sabin OPV2, with a modest decrease in plaque size (Fig. 1b, c). These initial properties suggested nOPV2-CD might demonstrate growth properties similar to traditional Sabin OPV2, and that the combination of codon-deoptimization within the capsid paired with the S15 domain V modification within the 5′ UTR did not over-attenuate the virus.

**Fig. 2:**
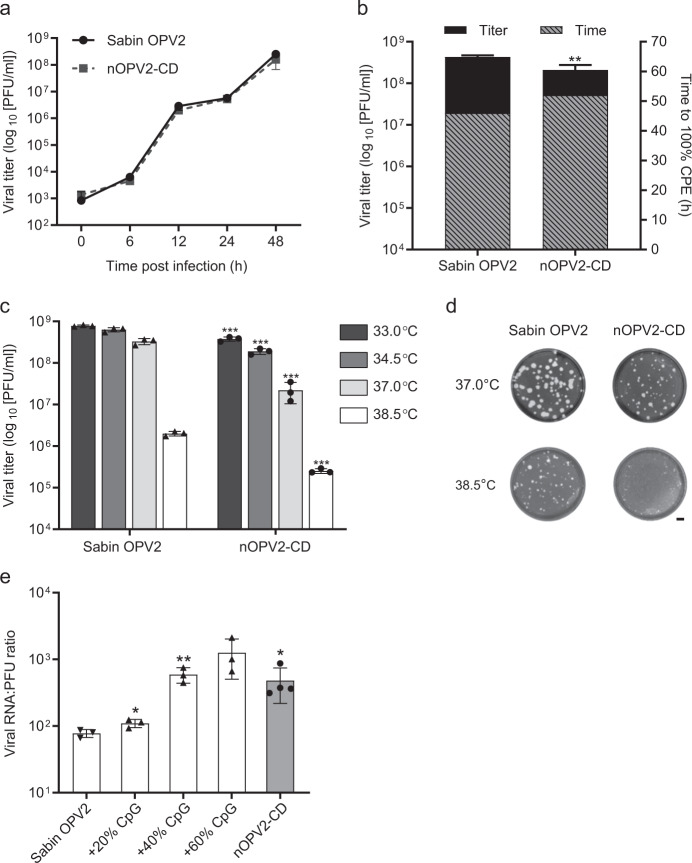
nOPV2-CD exhibits similar growth properties and temperature sensitivity as Sabin OPV2 but delivers more RNA per PFU. Vero cells were infected with Sabin OPV2 or nOPV2-CD at an MOI of 0.1. **a** Viral titers were determined at the indicated times post-infection at 33 °C via plaque assay. **b** Time to reach 100% cytopathic effect (CPE) at 33 °C and the corresponding viral titers at that time were determined via plaque assay. **c** Viral titers were measured via plaque assay after incubation of infected Vero cells at the indicated temperatures for 48 h. **d** Plaque morphology on Vero cell monolayers after 65 h. Scale bar = 1 cm. **e** Ratio of viral RNA copy number to PFU was established via qRT-PCR and plaque assay. Error bars indicate mean ± SD. *n* = 3 to 4 biologically independent replicates showing similar results. All *P* values were determined by unpaired two-tailed *t*-tests. *P ≤ 0.05, **P ≤ 0.01, ***P ≤ 0.001.

Growth of nOPV2-CD and Sabin OPV2 was compared under conditions mimicking those used in vaccine manufacturing. Vero cells infected at an MOI of 0.1 produced nearly equivalent titers of both vaccine strains throughout a 48-hour time course at 33 °C (Fig. 2a). Additionally, the time required for nOPV2-CD to reach 100% cytopathic effect (CPE) in Vero cells, as well as the resulting viral titer at 100% CPE, was comparable to that of Sabin OPV2 (Fig. 2b). These data showed that the growth properties of nOPV2-CD are favorable for vaccine production, suggesting that nOPV2-CD, like Sabin OPV2, can be manufactured at sufficient levels for supply demands and at an acceptable cost.

Temperature sensitivity is a hallmark of many successful live-attenuated vaccines and was historically a criterion to confirm attenuation of Sabin OPV vaccine stocks prior to release by manufacturers^40^. The temperature-sensitivity growth profile of nOPV2-CD mirrored that of Sabin OPV2 in Vero cells, with replicative fitness declining as temperature was increased from 33 °C to 38.5 °C (Fig. 2c, d). nOPV2-CD did exhibit titers approximately one log lower than Sabin OPV2 and modestly smaller plaque size at 37 °C, suggesting that replication under physiologic conditions (e.g., in the human gut) might offer enhanced attenuation. Interestingly, specific infectivity of nOPV2-CD was lowered, with over six-fold more copies of viral RNA delivered per PFU for nOPV2-CD versus Sabin OPV2 (Fig. 2e). Previous work has demonstrated similar trends for other codon-deoptimized polioviruses, with specific infectivity declining as the number of synonymous codon replacements increases^20,21^.

### Structural antigenicity of nOPV2-CD parallels that of Sabin OPV2

**Fig. 3:**
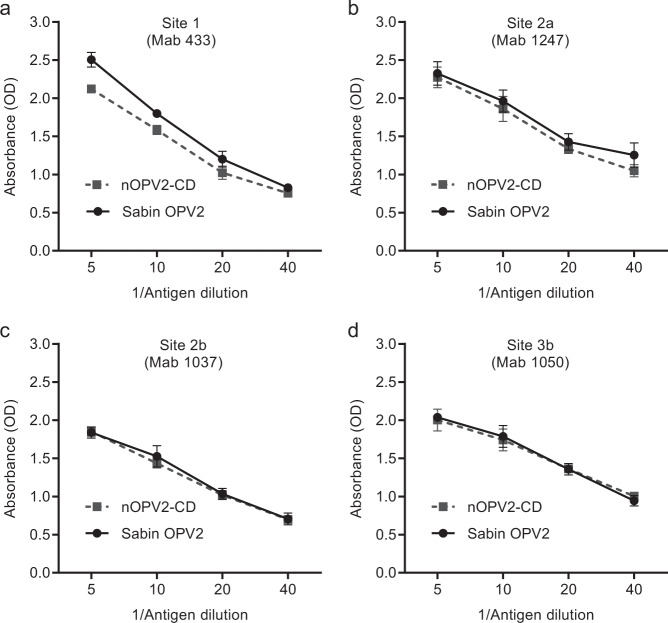
The antigenicity profile of nOPV2-CD parallels that of Sabin OPV2. The antigenicity of Sabin OPV2 and nOPV2-CD was determined via non-competitive sandwich ELISA using monoclonal antibodies specific for native structural conformations of four key antigenic sites on the virion particle. Dose-dependent reactivity to these four antibodies correlates with antigenicity. **a** Antigenic site 1 (MAb 433). **b** Antigenic site 2a (MAb 1247). **c** Antigenic site 2b (MAb 1037). **d** Antigenic site 3b (MAb 1050). Error bars indicate mean ± SD. *n* = 3 biologically independent replicates showing similar results.

A panel of monoclonal antibodies (MAb) with known reactivity to native structural conformations of four key antigenic sites (sites 1, 2a, 2b, and 3b) on the surface of polio virions was used in an ELISA to assess the antigenicity of nOPV2-CD (Fig. 3a–d). The dose response curve of nOPV2-CD paralleled that of Sabin OPV2, suggesting both strains are highly antigenic. The similarity in antigenicity results is perhaps expected, since nOPV2-CD should be structurally indistinguishable from Sabin 2 as only synonymous substitutions were introduced into the capsid region of nOPV2-CD.

### nOPV2-CD elicits comparable levels of neutralizing serum antibodies as Sabin OPV2 in a mouse model

**Fig. 4:**
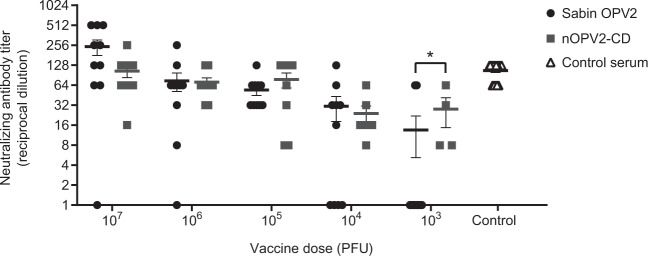
nOPV2-CD elicits a dose-dependent immune response comparable to that of Sabin OPV2. Juvenile interferon-receptor knockout mice expressing the human poliovirus receptor were inoculated i.p. with either Sabin OPV2 or nOPV2-CD at a range of doses. Neutralizing antibody titers were determined in sera at day 21 post-infection. Human sera collected from a polio-immunized individual served as a positive assay control. Titers for individual animals are shown, with error bars indicating standard error of the mean (SEM). *n* = 10 weight- and sex-matched mice per group. All *P*-values were determined by a two-tailed Mann-Whitney *U*-test. *P = 0.0230.

A transgenic mouse model has been developed that allows initial preclinical assessment of immunogenic potential^41^. Poliovirus can elicit a serum neutralizing antibody response in a dose-dependent manner in juvenile mice expressing the human poliovirus receptor (CD155) and deficient for the interferon-αβ receptor. Importantly, seroconversion in this particular model is dependent on replication in the animals, which also serves as a surrogate measure of infectivity. Mice were inoculated intraperitoneally with a range of doses of either Sabin OPV2 or nOPV2-CD and sera were collected after 21 days. Neutralizing antibody titers were determined via standard neutralization assay on HeLa cells (Fig. 4). The results show that nOPV2-CD elicited a dose-dependent neutralizing antibody response that was comparable to that of Sabin OPV2, providing preclinical evidence that nOPV2-CD replicated effectively, presented antigen, and elicited an antibody response.

### nOPV2-CD is attenuated in a mouse model of neurovirulence

In addition to being immunogenic, candidate nOPV2 strains should maintain at least a similar level of attenuation of neurovirulence as Sabin OPV2. Traditional Sabin OPV2, without any reversion mutations, has an acceptable low level of neurovirulence in animal models^42,43^ and is regarded as safe in humans. To assess the attenuation phenotype of nOPV2-CD, mice expressing the poliovirus receptor were inoculated intraspinally with stocks of nOPV2-CD or Sabin OPV2 and observed for signs of paralysis for up to 14 days. The dose required to paralyze 50% of the mice (PD_50_) was calculated using the Spearman-Karber method^44^. The PD_50_ for Sabin OPV2 in this model was 5.6 log_10_ CCID_50_, while nOPV2-CD generated a PD_50_ of 6.3 log_10_ CCID_50_ (Table 1). These results suggest that nOPV2-CD is slightly less neurovirulent than Sabin OPV2, fulfilling the attenuation criterion.

**Table 1:** nOPV2-CD is attenuated in a mouse neurovirulence model.

Virus	PD_50_ [log_10_ CCID_50_ (95% CI)]^a^	
	Passage 0	Passage 10
MEF-1	0.8 (0.3–1.3)	ND
Sabin OPV2	5.6 (5.0–6.2)	2.1 (1.6–2.6)
nOPV2-CD	6.3 (5.9–6.8)	5.4 (5.0–5.8)

*ND* not determined.

^a^Dose (as measured by CCID_50_) required to paralyze 50% of mice (PD_50_) was calculated using the Spearman-Karber method and 95% CI reported. *n* = 8 weight- and sex-matched mice per group.

### nOPV2-CD is more genetically stable than traditional Sabin OPV2

**Fig. 5:**
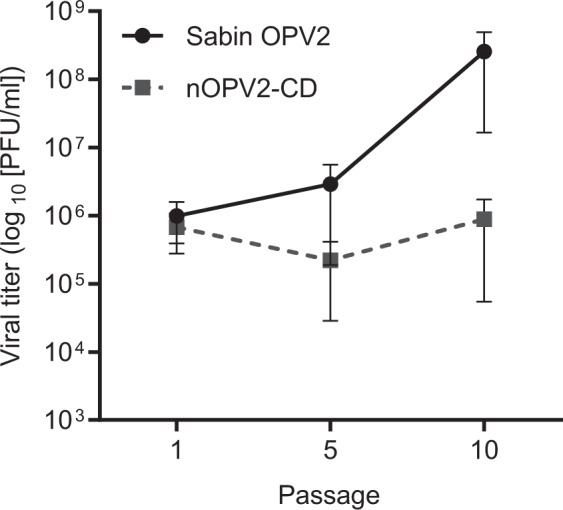
Growth of nOPV2-CD remains stable during serial passage in Vero cells. Vero cells were infected with Sabin OPV2 or nOPV2-CD at an MOI of 0.1 and incubated at 37 °C. After 10 h, virus was harvested, viral titers determined via plaque assay, and the amount of virus necessary to infect a subsequent round of Vero cells at MOI 0.1 calculated. A total of ten serial passages was completed, with the mean viral titer at the indicated passages shown. Error bars indicate mean ± SEM. *n* = 3 biologically independent replicates showing similar results. Viral stocks were derived from the tenth serial passage in Vero cells and used as inoculum for mouse neurovirulence testing (Table 1).

Greater genetic stability is a key goal in redesigned nOPV, to better safeguard against vaccine-associated paralytic poliomyelitis (VAPP) and emergence of VDPV arising from revertant vaccine strains. While no true surrogate exists to model viral replication within the human gut, serial passage of poliovirus in cell culture under conditions that increase selective pressure can mimic key virus genetic changes and phenotypic reversion observed in Sabin OPV recipients. To assess genetic stability, Sabin OPV2 and nOPV2-CD were serially passaged for 10 rounds in Vero cells at 37 °C and a low MOI of 0.1 (Fig. 5). Over ten passages, Sabin OPV2 steadily increased in titer, a likely indication of accumulating adaptive mutations. In comparison, titers of nOPV2-CD remained relatively constant throughout passaging.

To determine whether attenuation was maintained following passage, neurovirulence in a mouse model was evaluated using 10^th^ passage materials (Table 1). Upon passaging, Sabin OPV2 became more neurovirulent, with a 3.5 log drop in the amount of virus necessary to paralyze 50% of mice (from 5.6 to 2.1 log CCID_50_). In contrast, the attenuation of nOPV2-CD remained constant even upon passaging, with less than a 1 log difference in PD_50_ values between passaged and unpassaged virus. To assess whether this stability was also reflected at the genetic level, deep sequencing of the viral genome was performed on 10^th^ passage materials (Table 2). No changes were observed within the modified region of the 5′ UTR nor any of the 87 CpG nucleotide changes engineered within the codon-deoptimized capsid, demonstrating that the engineered design features were remarkably stable. In contrast, Sabin OPV2 reverted at the key 5′ UTR residue (nt 481) known to be the main “gate keeper” reversion mutation associated with increased neurovirulence in the majority of cVDPV2s. Change at a second well-known hotspot for reversion at amino acid position 143 in capsid protein VP1 was not observed during this particular passage of Sabin OPV2. However, changes at that position have been well documented following passage of Sabin OPV2 strains through the gut and in cell culture^18,34,36^. A 5′ UTR change at nucleotide 156 was detected at low levels (7.5%) in passaged nOPV2-CD, but this change did not significantly affect neurovirulence in the mouse model and is furthermore rarely associated with known VDPV2 sequence changes. A mutation in the viral 3 C protease region, present in both passaged strains, accumulated to a much higher level in Sabin OPV2 (77.1%) than nOPV2-CD (11.3%), and is likely an adaptive mutation to replication in Vero cells.

**Table 2:** Genetic stability of viruses following serial passage in Vero cells.

Viral gene	Nucleotide position^a^	Variant	Coding impact	Variant percentage^b^	
				Sabin OPV2	nOPV2-CD
None (5′ UTR)	156	T to C	None	—	7.5
None (5′ UTR)	481	A to G	None	18.6	—
VP1	2797	T to A	S106T	13.3	—
3 C	5774	A to T	Y113F	77.1	11.3

^a^Position designation based on Sabin-2.

^b^Variants greater than 5% reported.

## Discussion

A safer, more genetically stable, live attenuated poliovirus vaccine is critically needed for the poliovirus eradication endgame. Currently, more cases of poliomyelitis are caused globally by VDPVs than WPV and continued use of Sabin-based OPV has seeded additional VDPV outbreaks. Despite the withdrawal of Sabin OPV2, outbreaks have continued to occur in high-risk areas due to circulating type 2 VDPVs present prior to the switch, accidental use of trivalent OPV stocks, and deployment of a global stockpile of monovalent Sabin OPV2 in response to cVDPV2 outbreaks^12–15^. The latter inherently poses additional risk by reintroducing OPV2 into vulnerable populations^8^. With the inherent inability to interrupt transmission, IPV alone is not the solution.

To rationally design a new OPV candidate, we sought to further attenuate Sabin OPV2 using a two-pronged approach to target the viral capsid via codon deoptimization and the 5′ UTR via stabilization of known reversion hotspots, while still maintaining sufficient replicative fitness and immunogenicity. Yield of nOPV2-CD under conditions mimicking those used in vaccine production settings was similar between nOPV2-CD and Sabin OPV2, supporting the notion that nOPV2-CD could be manufactured at a similar cost. As measured by reactivity with a panel of monoclonal antibodies specific to key antigenic residues on the poliovirus surface, the antigenicity profile of nOPV2-CD was virtually indistinguishable from that of Sabin OPV2. This reflects the unmodified antigenic features that remain on the surface of the nOPV2 virion due to the silent nature of the engineered CD and 5′ UTR modifications. Remarkably, despite numerous nucleotide substitutions, virus production is only minimally reduced and antigenic structures are maintained. Using a transgenic mouse model to assess the capacity of vaccination to induce a neutralizing antibody response, nOPV2-CD demonstrated immunogenicity in a dose-dependent manner comparable to that of Sabin OPV2. Together with the antigenicity results, these data suggest that nOPV2-CD may elicit a protective immune response comparable to that of Sabin OPV2 in human recipients. Furthermore, since seroconversion in this animal model depends on replication^45^, the immunogenicity results also demonstrate that nOPV2-CD is replication competent and the engineered modifications do not over-attenuate the virus.

The design features of OPV2-CD were engineered to be inherently stable. Extensive mutation would be required to produce significant reversion of the 87 introduced CpG modifications. Importantly, the nOPV2-CD modified capsid cannot be lost by recombination with another poliovirus or a non-polio enterovirus (as frequently occurs with cVDPVs), safeguarding against propagation of unmodified capsid. Genetic stability of CD poliovirus is maximized by distributing nucleotide substitutions over the entire capsid coding region, which would require multiple mutations to produce significant reversion. Further, improved genetic stability of the 5′ UTR was engineered via inclusion of the S15 domain V modification^46^. This modification eliminates the ability of the virus to revert by single nucleotide changes within the main Sabin OPV2 attenuation determinant, which otherwise occurs quickly in the human gut following oral delivery. To assess preclinical genetic stability, we used serial cell culture passage at low multiplicity of infection and high temperature to model selective pressure in the human gut. While Sabin OPV2 reverted at the key neurovirulence determinant within the 5′ UTR, all of the engineered genetic modifications within OPV2-CD remained stable. The attenuated phenotype of passaged nOPV2 also was maintained. In stark contrast, Sabin OPV2 became significantly more neurovirulent upon passage, phenotypically resembling vaccine-derived poliovirus.

The results from this preclinical evaluation of nOPV-CD informed the decision to initiate the first in-human clinical trial of new polio vaccines since the 1950s. A manuscript describing the results of the initial Phase I testing of our nOPV-CD candidate as well as an additional candidate was recently published^38,39^. In that study, 15 adults with routine IPV immunization backgrounds received 10^6^ 50% cell culture infectious dose units (CCID_50_) of nOPV2-CD by oral administration. At 28 days post-vaccination, a greater than 12-fold median increase in titers of serum neutralizing antibodies against type 2 poliovirus was detected. All subjects had seroprotective antibody titers post-vaccination, with a seroconversion rate (≥4-fold rise in titer) of >84%, suggesting that nOPV2-CD was viable within the human gut and elicited an immune response. Shedding of nOPV2-CD in stool was detected from 13 out of 15 vaccinees. Interestingly, the duration and magnitude of shedding of nOPV2-CD was less than a second candidate nOPV strain evaluated during the same trial, as well as what has been observed in the past for Sabin OPV2. This may be a direct result of reduced replicative fitness bestowed by the codon-deoptimized capsid within the gut. In addition, the delivery of a larger payload of pathogen-associated molecular material may potentially enhance the host antiviral immune response within the gut of nOPV2-CD vaccine recipients, leading to lower levels of shedding. Indeed, the host response to codon-deoptimized picornaviruses is not identical to that of prototype strains^28,47,48^. Our ongoing work seeks to better define the underlying mechanisms of attenuation by codon deoptimization. While differences in specific infectivity do exist between nOPV2-CD and Sabin 2, D-antigen content has subsequently been found to be nearly identical, which has provided confidence that comparable levels of immune-stimulating viral particles are present. Supportive of the enhanced genetic stability observed preclinically, virus shed and recovered from vaccinees maintained the attenuated phenotype as evaluated by the mouse neurovirulence assay. Additionally, analysis of the viral genome from shed virus demonstrated that all genetic modifications engineered into nOPV-CD were stably maintained after replication in humans^39^.

The results of this initial clinical trial demonstrated that nOPV-CD is viable, immunogenic and safe in human subjects. Additionally, they provided further evidence that nOPV-CD is more stable with respect to the attenuation phenotype than traditional Sabin OPV2. A larger Phase II study in adults, toddlers, and infants is currently underway, which will provide more robust safety, immunogenicity, and genetic stability data. The main purpose of nOPV will be to support the Global Polio Eradication Initiative during the final push to eliminate poliovirus globally, with the most immediate need in ongoing outbreak settings to control and eliminate cVDPVs. In addition, in the event that current immunization programs relying on IPV and bivalent OPV fail to eradicate polio, nOPV may also serve as a “back-up” vaccine tool that induces the mucosal immunity necessary to prevent transmission, without the risk of cVDPV emergence. Not only does nOPV-CD have the potential to be a key player in the success and long-term maintenance of polio eradication, but it also exemplifies new concepts in vaccine development.

## Methods

### Virus and cells

Production of prototype Sabin 2 and MEF-1 infectious clones has been described previously^20,21^. The Sabin Original + 2 type 2 OPV seed strain was provided by R. Mauler of Behringwerke AG (Marburg, Germany). The MEF-1 type 2 IPV seed strain was a gift of Connaught Laboratories (Toronto, Ontario, Canada), which was received in 1960 as their lot 55. Briefly, the prototype Sabin 2 (GenBank DQ205099) and MEF-1 (GenBank CS406482) clones were produced from full-length PCR products of cDNAs derived from the source viruses and cloned into pUC19 using standard restriction enzymes and T4 DNA ligase (New England Biolabs). Ligated DNA was transformed into SoloPack Gold Supercompetent Cells (Agilent). The clones were designed with 19 bases of the T7 promoter (TAATACGACTCACTATAGG) immediately upstream of the 5′ end of the viral sequence and a 30-base polyA tail after the 3′ end, followed by a HindIII restriction endonuclease site used for linearizing the plasmids. RD cells (ATCC CCL-136) were used for transfection of infectious clones and virus stock expansion. Monolayers of HeLa cells (ATCC CCL-2) were used for plaque assays. Vero cells (ATCC® CCL-81) were used for plaque, growth, and stability assays.

### Codon deoptimization of poliovirus clones

To codon-deoptimize poliovirus, we engineered the codons for serine, proline, threonine, and alanine in the capsid region to the synonymous versions that contain CpG in the second and third positions of the codon: UCG, CCG, ACG, and GCG. These codons are disfavored in the poliovirus genome. The CpG-containing codons were introduced at 20, 40, 60, 80 or 100% of the possible sites within the capsid region of Sabin 2 and MEF-1 clones. Saturation of the CpG incorporation was spread evenly over the capsid cassette. The S15 domain V 5′ UTR modification has been described elsewhere^38,46^. Standard cloning methods were used to insert the modified 5′ UTR S15 domain V and capsid constructs (synthesized by GenScript)^20^. The nOPV2-CD clone contains the 40% CpG-modified codon-deoptimized capsid cassette and the 5′ UTR S15 domain V insert. The full genome sequence of nOPV-CD can be found in GenBank (accession number MN654096).

### Virus preparation

Virus stocks were produced with minor modifications of our published methods^20^. In vitro transcripts of viral RNA produced with a MEGAscript Kit (Life Technologies) were transfected into 80% confluent RD cell monolayers using a TransIT-mRNA Transfection Kit (Mirus). Sabin OPV2 and nOPV2 cultures were incubated at 34.5 °C and MEF-1 at 37 °C. Complete cytopathic effect (CPE) was observed for most viruses after one to two days, although the CPE in the most attenuated viruses did not proceed to completion. Depending on the CPE level of the transfections, 200 to 500 μl from each well was transferred to a confluent RD cell monolayer in a 75-cm^2^ flask containing complete minimal essential medium (MEM). Flasks were incubated until complete CPE was observed, or to a maximum of 72 h. The sequences of all virus stocks were verified by PCR amplification of large overlapping fragments and Sanger sequence analysis.

### Plaque assay and plaque size measurement

Plaque assays were performed as described previously, with minor modifications^20^. Briefly, confluent HeLa or Vero cell monolayers in 10-cm cell culture dishes were washed, inoculated with virus in MEM, and incubated at room temperature for 30 min prior to the addition of 0.45% SeaKem LE agarose (Lonza) in MEM containing 2% fetal bovine serum (FBS). Plates were incubated for 65 h, fixed and stained with 4.4% formaldehyde/0.05% crystal violet. Plaque sizes were quantified from digital images of the plates made with a Bio-Rad Molecular Imager Gel Doc XR^+^ Imaging System and by subsequent image analysis using MATLAB Image Processing Tool v2.8.

### Viral growth assay

Virus was added to confluent monolayers of Vero cells at the indicated MOI for 30 min at room temperature. Inoculum was removed and the cells washed once before adding complete MEM containing 2% FBS. The 0 h sample was frozen immediately, while additional time points were incubated at the indicated temperatures. Cells were frozen and thawed a total of three times before centrifugation at 15,000*g* for 15 min at 4 °C. Supernatants were collected and virus titered by plaque assay on HeLa cells.

### Determination of ratios of viral RNA to PFU

Viral RNA present in clarified virus stocks was measured by quantitative, reverse-transcription polymerase chain reaction (PCR) using Applied Biosystems 7500 Real-Time PCR System. Virus stocks were treated with RNase Cocktail (Ambion) for 30 min at 37 °C before adding TRizol LS (Invitrogen) to extract RNA per manufacturer’s instructions. RNA was further purified using Direct-zol RNA MiniPrep Plus column (Zymo), also according to the manufacturer’s instructions. Eluted RNA was reverse-transcribed using random hexamers and SuperScript IV reverse transcriptase (Invitrogen). To determine genome copy number, the resulting cDNA was used as template in a quantitative PCR assay using primers 78F (CGCCTGTTTTATACTCCC) and 324R (CTCATCAGCCTAAGCTAC) to amplify a region within the 5′ UTR, and amplicon yield was measured via TaqMan probe 184P (GCACTTCTGTTTCCCCGGTG). Serial dilutions of a linearized plasmid DNA were used as standards to calculate molecular copy number. Viral titers corresponding to the same virus stocks were determined by HeLa cell plaque assay at 37 °C, as described above.

### Antigenicity assay

A non-competitive sandwich ELISA assay was used to measure reactivity with monoclonal antibodies (MAbs) specific for four different antigenic sites present on native virus particles^49^. Two-fold serial dilutions of antigen were captured with an in-house serotype-specific rabbit polyclonal antibody (diluted 1:500), then detected using a panel of in-house serotype-specific monoclonal antibodies (diluted 1:100) produced from hybridomas, followed by anti-mouse IgG peroxidase conjugate (Sigma-Aldrich via Merck Cat No. A6782, diluted 1:400). The in-house panel consisted of MAbs specific for native conformations of antigenic sites 1 (MAb 433), 2a (MAb 1247), 2b (MAb 1037), and 3b (MAb 1050), which were used as primary antibodies. The reactivity of each test sample was evaluated against a Sabin 2 reference.

### Mouse immunogenicity assay

Poliovirus receptor (PVR)-transgenic (Tg21), type I interferon receptor knockout (IFNR-KO) mice (PVRTg21-IFNR-KO) were kindly provided by Dr. Satoshi Koike and maintained in the AAALAC-certified animal facility at UCSF. All animal experiments were conducted in accordance with the guidelines of the Laboratory Animal Center of the National Institutes of Health. The Institutional Animal Care and Use Committee of University of California at San Francisco approved all animal protocols (approved protocol no. AN128674-03A).

Four-week-old PVRTg21-IFNR-KO mice were inoculated with various amounts of virus (5 male and 5 female mice per dose) via the intraperitoneal route. Blood samples were collected at day 21 post-immunization from the retro-orbital sinus. Human sera collected from a polio-immunized individual served as a positive assay control.

### Virus neutralization assay

Mouse serum samples were diluted 4-fold with PBS and inactivated at 56 °C for 30 min. The inactivated sera were serially diluted 2-fold with diluent (DMEM/F12 with 1% BSA and 1× penicillin/streptomycin), mixed with an equal volume of OPV2 (calculated to deliver 100 CCID_50_ of virus), and incubated for 2 h at 33 °C. Control serum was treated the same as mouse serum samples. Subsequently, 100 µl of the serum/virus mixture was transferred to monolayers of HeLa cells in 96-well plates and incubated at 33 °C for 7 days. The plates were then fixed with 1% formaldehyde, stained with 1% crystal violet and examined for CPE to determine antibody titer. The titer of neutralizing antibody was determined as the highest dilution that prevented the development of CPE.

### Serial passage in Vero cells for genetic stability assays

Monolayers of Vero cells were infected with virus at an MOI of 0.1 at 37 °C. After 10 h (approximately a single cycle of replication), virus was harvested, titered by plaque assay, and used for subsequent rounds of infection at MOI of 0.1. Ten serial passages proceeded in this manner. Virus was deep sequenced at the 10^th^ passage and used for subsequent mouse neurovirulence assays.

### Mouse neurovirulence test: intraspinal inoculation of Tg66-CBA mice

Transgenic mice expressing the human poliovirus receptor (Tg66-CBA) were inoculated by the intraspinal route (i.s.) with 10-fold serial dilutions of Sabin 2 and nOPV2-CD. The dose (CCID_50_) required to paralyze 50% of the mice (PD_50_) was calculated using the Spearman-Karber method^44^. Intraspinal inoculation was performed essentially according to the standard operating procedure available from WHO^42^. The adaptation of the protocol included using higher doses and fewer mice per dose. Additionally, Tg66-CBA mice (which also express the human poliovirus receptor)^46^ were used in substitution for the TgPVR21 strain used in the WHO assay. Both strains have similar sensitivities to Sabin 2 when inoculated by the i.s. route (Macadam, unpublished). Briefly, Tg66-CBA mice (6–8 weeks old) in groups of 8 (weight and sex-matched) were sedated and inoculated into the lumbar region of the spinal cord with 5 µl of each dose and observed for occurrence of paralysis for up to 14 days. Mice with paresis/paralysis were scored positive and mice surviving for 14 days with no clinical signs were scored negative.

The Tg66-CBA mouse experiments at NIBSC were performed under Home Office licenses PPL 80/2478 and PPL 70/8979 granted by the UK Home Office under the Animal (Scientific Procedures) Act 1986 revised 2013 and reviewed by the internal NIBSC Animal Welfare and Ethics Review Board before submission.

### Deep sequencing of poliovirus genomic material

Viral RNA was extracted from 140 µl of cell culture supernatants using QIAamp viral RNA mini kit (QIAGEN). Extracted RNA was eluted in a final volume of 60 µl of sterile RNase-free water and frozen at −80 °C until further use. cDNA was synthesized using SuperScript III Reverse Transcriptase Reagent Kit and primers binding at the 3′-end of viral RNA as described^50^. Full-length PCR amplification of viral cDNA was performed by using the PCR SuperMix High Fidelity kit (Invitrogen) with primers described previously^50^. Veriti Thermal Cycler (Applied Biosystems) was used to perform the PCR at the following conditions: incubation for 2 min at 94 °C, followed by 30 cycles, each consisting of 10 sec at 94 °C, 30 sec at 64 °C, and 8 min at 68 °C, followed by the final extension step for 7 min at 68 °C.

PCR product was analyzed by electrophoresis in a 1% agarose gel, and then purified using Agencourt AMPure XP Reagent (Beckman Coulter). Purified DNA was eluted in 50 µl of DEPC-treated water. DNA concentration was measured with Qubit 2.0 Fluorometer (Invitrogen by Life Technologies), and the DNA frozen at −20 °C for further use. Illumina libraries were prepared using Nextera XT Library Prep Kit (Illumina) and Nextera XT Index Kit [24 indexes, 96 samples] (Illumina). Library quality was assessed on a 4200 TapeStation Bioanalyzer Instrument (Agilent Technologies) using High Sensitivity D1000 Reagents (Agilent Technologies) and High Sensitivity D1000 ScreenTape (Agilent Technologies). Deep sequencing was performed using a MiSeq instrument (Illumina) using MiSeq Reagent Kit v2, 500-cycles (Illumina). Bioinformatic analysis was performed using in-house SWARM and HIVE software^51^. The pipeline included quality controls to remove sequence information with phred scores below 30 and removal of adapter and index sequences, followed by alignment of sequence reads to the reference viral sequence, and computation of the profile of sequence heterogeneities^52,53^. The depth of sequencing coverage ranged between 1000 and 5000 reads per nucleotide, meaning that the quantification precision was between 0.02 and 0.1%. Background determined by sequencing viruses derived from homogeneous DNA plasmid ranged between 0.01% and 0.12%, therefore the lower limit of quantification was within the 0.2% range.

### Statistical analyses

Data were prepared and analyzed with Prism 8 (GraphPad) using statistical methods described below. Statistical significance was set as *P*-value ≤ 0.05 (*P-value ≤ 0.05; **P-value ≤ 0.01; ***P-value ≤ 0.001). (Fig. 2a) An unpaired two-tailed *t*-test was performed. No statistically significant differences were detected between Sabin OPV2 and nOPV2-CD at any time. (Fig. 2b) An unpaired two-tailed *t*-test was performed. Significant differences were detected between Sabin OPV2 and nOPV2-CD viral titers (*P* = 0.0076). (Fig. 2c) An unpaired two-tailed *t*-test was performed. Significant differences were detected between Sabin OPV2 and nOPV2-CD at all temperatures: 33 °C (*P* = 0.0003), 34.5 °C (*P* = 0.0006), 37 °C (*P* = 0.0007), 38.5 °C (*P* = 0.0003). (Fig. 2e) An unpaired two-tailed *t*-test was performed. Significant differences were detected between Sabin OPV2 and Sabin OPV2 + 20% CpG (*P* = 0.0419), Sabin OPV2 + 40% CpG (*P* = 0.0044), and nOPV-CD (*P* = 0.0487). There was not a statistically significant difference between Sabin OPV2 and Sabin OPV2 + 60% CpG (*P* = 0.0536). (Fig. 3a–d) An unpaired two-tailed *t*-test was performed. No statistically significant differences were detected between Sabin OPV2 and nOPV2-CD. (Fig. 4) A two-tailed Mann-Whitney U test was performed. No significant differences were detected between Sabin OPV2 and nOPV2-CD immunogenicity at the 10^7^ (*P* = 0.154), 10^6^ (*P* = 0.7236), 10^5^ (*P* = 0.4985), or 10^4^ doses (*P* = 0.8461). At the 10^3^ dose, a significant difference was detected, with nOPV2-CD immunogenicity being higher than that of Sabin OPV2 (*P* = 0.0230). (Fig. 5) An unpaired two-tailed *t*-test was performed. No statistically significant differences were detected between Sabin OPV2 and nOPV2-CD. (Table 1) The dose required to paralyze 50% of the mice (PD_50_) was calculated using the Spearman-Karber method^44^ with 95% confidence intervals (CI) reported.

### Reporting Summary

Further information on research design is available in the Nature Research Reporting Summary linked to this article.

## Supplementary information

Reporting Summary

## Data Availability

The data that support the findings of this study are available from the corresponding author upon request. The full genome sequence of nOPV2-CD can be found in GenBank (accession number MN654096).
